# Identification of *Bacillus* strains by MALDI TOF MS using geometric approach

**DOI:** 10.1038/srep16989

**Published:** 2015-11-23

**Authors:** Konstantin V. Starostin, Evgeny A. Demidov, Alla V. Bryanskaya, Vadim M. Efimov, Alexey S. Rozanov, Sergey E. Peltek

**Affiliations:** 1 Institute of Cytology and Genetics, The Siberian Branch of the Russian Academy of Sciences, Novosibirsk, 630090, Russian Federation

## Abstract

Microorganism identification by MALDI TOF mass-spectrometry is based on the comparison of the mass spectrum of the studied organism with those of reference strains. It is a rapid and reliable method. However, commercial databases and programs are mostly designed for identification of clinically important strains and can be used only for particular mass spectrometer models. The need for open platforms and reference databases is obvious. In this study we describe a geometric approach for microorganism identification by mass spectra and demonstrate its capabilities by analyzing 24 strains belonging to the *Bacillus pumilus* group. This method is based on representing mass spectra as points on a multidimensional space, which allows us to use geometric distances to compare the spectra. Delimitation of microorganisms performed by geometric approach correlates well with the results of molecular phylogenetic analysis and clustering using Biotyper 3.1. All three methods used allowed us to reliably divide the strains into two groups corresponding to closely related species, *Bacillus pumilus* and *Bacillus altitudinis*. The method developed by us will be implemented in a Web interface designed for using open reference databases for microorganism identification. The data is available at http://www.bionet.nsc.ru/mbl/database/database.html.

When studying bacterial strains isolated from extreme environments, we require rapid and reliable identification of bacterial strains, including those of the genus *Bacillus*. The genus *Bacillus* contains Gram-positive aerobic or facultative anaerobic rod-shaped bacteria that form intracellular spores. It includes over 80 valid species^1^. Representatives of this genus are abundant in soil, air, and water, and are widely used as sources of industrial enzymes for food, textile and chemical industries^2^. They are also used as expression hosts for recombinant genes^3^, as well as a source of recombinant genes^4^. *Bacillus* strains are promising for agriculture as plant growth promoting rhizobacteria^5^ and for usage in decontamination systems^6^,^7^.

In the last 30 years systematics of the genus was substantially revised. Some species were isolated into new genera: *Alicyclobacillus*, *Paenibacillus*, *Aneurinibacillus*, *Brevibacillus*, *Halobacillus*, *Virgibacillus*, *Filobacillus*, and *Jeotgalibacillus*. Also, the genus *Bacillus* contains several closely related species group, whose delimitation is difficult. For example, the *Bacillus cereus* group includes *Bacillus cereus*, *Bacillus anthracis*, and *Bacillus thuringiensis*, which are genetically very similar, but nevertheless are considered separate species due to different pathegenicity^8^. Another example is the *Bacillus subtilis* group, which contains the species *Bacillus subtilis* subsp. *subtilis*, *Bacillus amyloliquefaciens*, *Bacillus licheniformis*, *Bacillus atrophaeus*, *Bacillus mojavensis*, *Bacillus vallismortis*, *Bacillus subtilis* subsp. *spizizenii*, and *Bacillus sonorensis*. The 16S rRNA gene sequences of these species have over 99% sequence similarity, so they cannot be distinguished based on it alone^9^. A new species, *Bacillus safensis*, was isolated from *B. pumilus* based on the sequence of the gyrB gene^10^, and three more species were described using the polyphasic taxonomy approach: *Bacillus altitudinis*, *Bacillus stratosphaericus*, and *Bacillus aerophilus*^11^. These species have closely related 16S rRNA gene sequences and form the *B. pumilus* group^12^.

Classic methods of microorganism identification, such as biochemical tests and DNA sequencing, are time-consuming and labor-intensive. The more recently developed approach that uses MALDI TOF mass-spectrometry is simple and rapid. It is based on the comparison of protein spectrum of the studied specimen to a database of reference spectra^13^. Several studies demonstrated high reproducibility of this method provided standard protocols are used^14^,^15^,^16^,^17^. Identification accuracy is known to tolerate varying growth conditions^17^,^18^,^19^.

Effective application of the mass-spectrometry approach requires a comprehensive reference database, as well as specific software for comparison of spectra. Several commercial platforms are currently available: Biotyper (Bruker Daltonicks), Saramis (Shimadzu), Microbelynx (Waters Corporation), and Andromas. These platforms are designed for particular models of mass spectrometers and are rather expensive. They are also oriented towards clinical diagnostics and contain predominantly pathogenic species and strains. Due to these limitation, research groups have to design their own “in house” databases, mathematical algorithms and software. Some commercial products, such as Statgraphics Plus 5.1 (Statpoint Technologies) and BioNumerics 6.0 (Applied-Maths) allow one to create “in house” databases, but open platforms including user-filled databases and software for mass spectra analysis are required for effective development of the field^20^.

One of the first attempts to create such a database was the BGP-database^21^ (http://bgp.sourceforge.net). Another example is the Spectra bank (http://www.spectrabank.org), which is a database of mass lists characteristic for species or strains. It currently contains about 200 specimens. Characteristic mass lists may be compared using SPECLUST^22^, which performs cluster analysis by building a dendrogram. However, it cannot do database searches and does not adjust for relative peak intensity.

Geometrical methods that represent source data as points in a multidimensional space are widely used for mass-spectrometry data. They are mainly applied in cluster analysis and graphical representation of breaking spectra into groups using such methods as PCA and MDS^23^,^24^. Metrical distances, such as Euclidean distances, can be used as criteria for comparing unknown spectra with databases. For example, the Hamming^23^ and Mahalanobis^25^ distances were proposed.

Since our main goal is to develop a platform for an open database for microorganism identification, the mathematical algorithms have to be simple and not calculation-intensive, but at the same time significantly precise. We used the following algorithm implemented using the JACOBI-4 program developed in ICiG SB RAS^26^: (1) A set of data represented as (*m*/*z*; *intensity*) is transferred on an (*x*; *y*) coordinate plane, where *x*_*i*_ are discrete with a specified interval, *y*_*i*_ are calculated for each *x*_*i*_ node according the peak curve formula, which allows one to represent the spectrum as a vector in multidimensional space and to calculate a centroid for set of vector for tested microorganisms. This transformation allows one to apply all geometric methods; (2) A matrix of Euclidean distances and Jaccard coefficients (JC) representing measures of spectra dissimilarity/similarity is calculated for the obtained vectors. The value of 1 − *JC* is a metric^27^, and is therefore suitable for our goals; (3) The Principal Coordinates method (PCo) and dendrogram construction was used to perform cluster analysis and data visualization.

The aim of this study was to test if the approach presented in this study could be used to identify closely related species. As an example we took a set of *B. pumilus* group strains that had over 98% sequence similarity for the 16S rRNA gene. For the 24 studied strains, we obtained mass spectra series and calculated centroids for cluster analysis. These data were compared with the results of 16S rRNA gene sequencing and MALDI Biotyper 3.1 program (Bruker Daltonics) analysis. The obtained centroids were used as a reference database for identification of two replicates of the studied strains. Identification was based on Euclidean distances and JC used as similarity measures.

## Results

### 16S rRNA analysis

**Figure 1:**
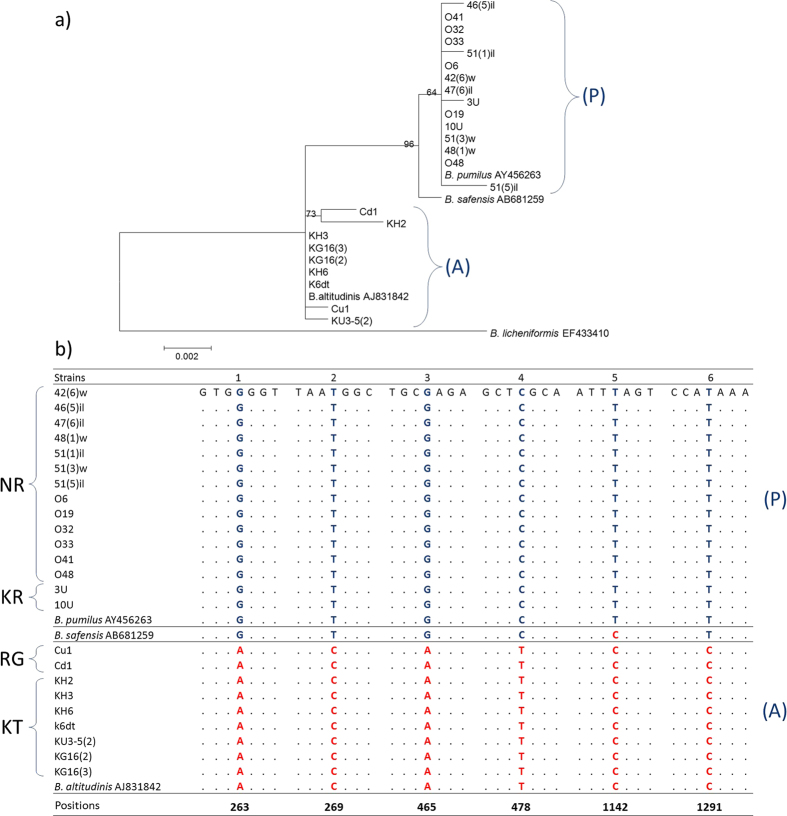
Phylogenetic analysis. (**a**) Phylogenetic tree constructed using the Maximum Likelihood algorithm for the 16S rRNA gene sequences. Numbers above branches indicate bootstrap support. Sequence distances represent the number of substitutions per 1000 nucleotides. The following type strains sequences were used for comparison: *B. pumilus* (AY456263), *B. safensis* (AB681259), and *B. altitudinis* (AJ831842); *B. licheniformis* (EF433410) was used as an outgroup. (**b**) Fragments of the alignment containing marker substitutions. Numbers in the bottom line indicate their positions in the *B. altitudinis* sequence (AJ831842).

Since we could not reliably identify the strains using the GenBank database (http://blast.ncbi.nlm.nih.gov/Blast.cgi) based on 16S rRNA gene sequences, we compared them to type strain sequences of the *Bacillus pumilus* group: *B. pumilus* (AY456263), *B. altitudinis* (AJ831842), and *B. safensis* (AB681259) (Fig. 1a). *B. stratosphaericus* and *B. aerophilus* were excluded from the analysis, because their 16S rRNA sequences were identical to *B. altitudinis* and these strains were absent from microbial strain catalogues. Two groups referred to as the A and P groups were detected in our dataset with the bootstrap support of 96. The A group included strains isolated from Kamchatka thermal springs (KT), rhizosphere of higher plants from the Novosibirsk oblast (RG), as well as the *B. altitudinis* type strain (AJ831842). The P group contained strains from saline lakes of the Novosibirsk oblast (NR), complex ore deposits of the Kemerovo oblast, and type strains of *B. pumilus* (AY456263) and *B. safensis* (AB681259). Within the groups, strains were closely related, except for the Cd1 and KH2 strains, which formed a single subcluster within the A group with the bootstrap support of 73. Visualization of the sequences in BioEdit allowed us to find six marker substitutions (Fig. 1b) that distinguished these clusters. Other substitutions were uninformative. Sequences of the 16S rRNA genes of the strains isolated from Kamchatka thermal springs (KT) and from the rhizosphere of higher plants from the Novosibirsk oblast (RG) were identical to *B. altitudinis* AJ831842, while those of the strains from saline lakes of the Novosibirsk oblast (NR) and complex ore deposits of the Kemerovo oblast (KR) did not differ from *B. pumilus* (AY456263). *B. safensis* (AB681259) differed by a single nucleotide substitution from *B. pumilus* (AY456263), which allowed us to identify NR and KR strains as *B. pumilus* (Fig. 1b). The KH2 and Cd1 strains had identical patterns of these six marker substitutions to the *B. altitudinis* (AJ831842) type strain and the rest of the A group. The sequence of *B. safensis* type strain (AB681259) differed from that of *B. pumilus* (AY456263) by one marker substitution. Based on this, we identified the representatives of the A group as *B. altitudinis*, and of the P group, to *B. pumilus*.

### Analysis of mass spectra

**Figure 2:**
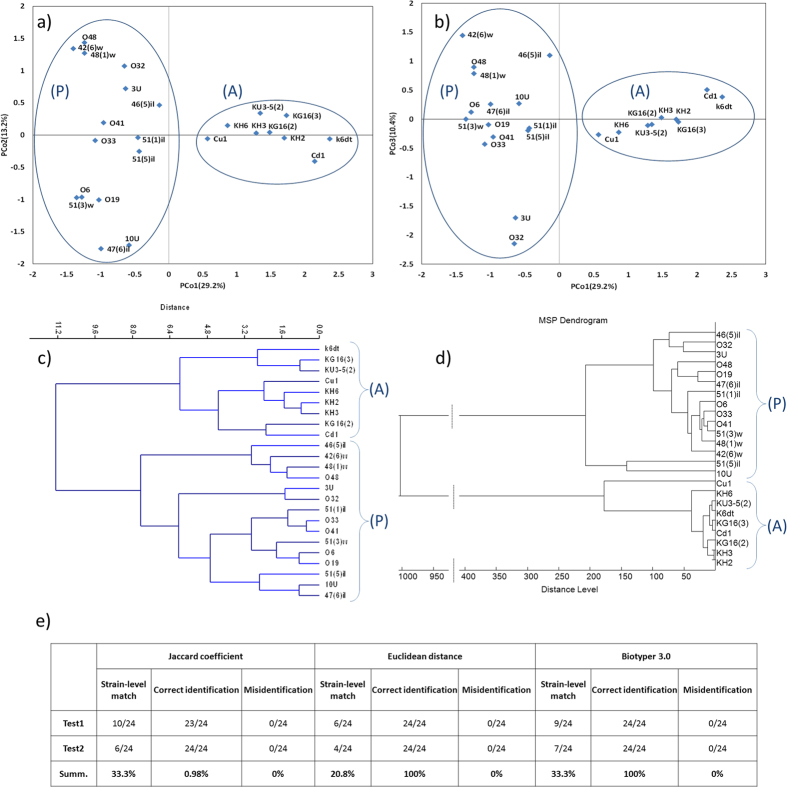
Phyloproteomic analysis. Position of spectra centroids on the principal coordinates plane: (**a**) PCo1, PCo2; (**b**) PCo1, PCo3. The proportions of the total variance for those axes were 29.2% for PCo1, 13.2% for PCo2, and 10.4% for PCo3, which sums up to 52.9%. The groups A and P are framed. Dendrograms constructed based on the distances among spectra centroids by the Ward’s method (**c**) and by MSPs clustering in Biotyper 3.1 (**d**). Strains were divided into two clusters that correspond to *B. altitudinis* (A) and *B. pumilus* (P); (**e**) Wet-lab experiment: identification of the studied centroids for two biological replicates using JC, Euclidean distances, and Biotyper 3.1 (cutoff criteria - 2.0, wich is defined Bruker as “secure genus identification, probable species identification”). (**e**) Wet-lab experiment: identification of the studied centroids for two biological replicates using JC, Euclidean distances, and Biotyper 3.1 (cutoff criteria - 2.0, wich is defined Bruker as “secure genus identification, probable species identification”). Strain-level match - case when centroid of tested specimen and closest centroid in data base belong to the one and the same strain.

Mass spectra were obtained and processed as described in the Materials and Methods section. For statistical significance 12 samples were taken for each strain, and three independent spectra were taken for each sample. Most of the obtained spectra contained 50 to 60 mass peaks in the 2–10 kDa range. The matrix of Euclidean distances among centroids, transformed using the principal coordinates analysis, was visualized as two 2-dimensional plots in the PCo1, PCo2 and PCo1, PCo3 coordinates (Fig. 2a,b). In both projections, the two groups differ by the position on the PCo1 axis that provides the largest amount of information. *B. altitudinis* strains fell into the A group, and *B. pumulus* strains, into the P group. Welsh’s t- test was used to verify the separation of strain centroids on the groups A or P (*t* = 11.16; *p* < 10^−6^; *df* = 22). The distance between the A and P sample centers along the PCo1 axis was 2.4, and standard deviations for these samples were 0.57 and 0.39, respectively. We should also note that the P group had higher variance along the PCo2 axes PCo3 in comparison to the A group, which indicates that it has higher heterogeneity. Cluster analysis was performed by constructing a dendrogram by the Ward’s method using all 23 coordinates obtained by transforming the matrix of Euclidean distances (Fig. 2c). A phyloproteomic dendrogram built using Biotyper 3.1 is shown for comparison (Fig. 2d). Both dendrograms demonstrate two clusters that correspond to the A and P groups on the plot (Fig. 2a,b) and the 16S rRNA gene tree (Fig. 1a).

**Figure 3:**
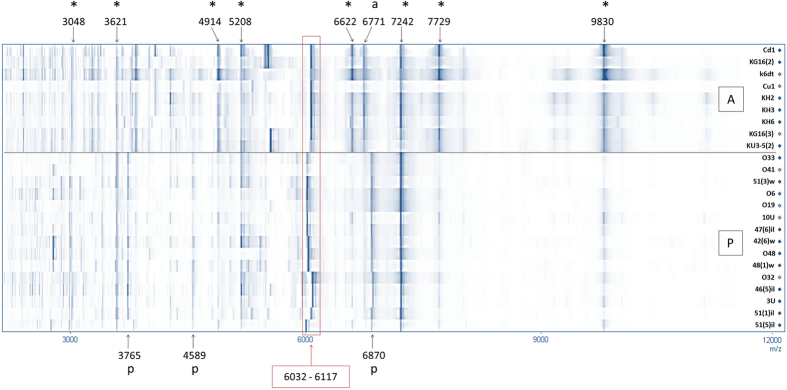
Gel view of averaged mass spectra. Common peaks are indicated by asterisks; peaks characteristic for the A group, by (a); peaks characteristic for the P group, by (p). A group of high-intensity peaks in the 6032–6117 Da range is framed.

We performed a SPECLUST analysis which yielded a list of common and group-specific peaks. Eight peaks were found in all studied strains : 3048, 3621, 4914, 5208, 6622, 7242, 7729, and 9830 Da. The A group was distinguished by the presence of the 6671 Da peak, while the P group had three characteristic peaks: 3765, 4589, and 6870 Da. Averaged mass spectra visualized in gel view demonstrate that the A and P groups differ by the presence of group-specific peaks, as well as by their relative intensities (Fig. 3). For example, the 4914, 6622, 7729, and 9830 Da peaks have higher intensities in the A group. Several high-intensity peaks at 6032, 6048, 6063, 6099, and 6117 Da demonstrated no group specificity. *B. altitudinis* strains had only the 6099 Da peak, while a *B. pumilus* strain may have any of these peaks. This is likely the cause of high dispersion along the PCo2 and PCo3 axes. We suggest that these peaks represent a single protein that is highly polymorphic in *B. pumilus* group.

Cross-validation was performed by two methods: by excluding one of each ten random sets of spectra samples and by excluding all spectra for a random strain. For the first method, spectra were correctly assigned to centroids in 96.7% of cases. Accuracy of group determination (A or P) was 99.9%.

The obtained centroids were used as a reference database and a training data set for calculating a cutoff criterion for identification of the studied strains. JC cutoff criterion value was 0.278 for the A group and 0.158 for the P group, and for the Euclidean distances, 3.35 and 3.01, respectively. We performed two independent wet-lab experiments for method verification. Figure 2e demonstrates results of strain verification using our reference database and Biotyper 3.1. Identification accuracy was 100% for Euclidean distances and 98% for JC. We should note that the single unidentified specimen was on the border of identification correctness; its score was 0.277, while cutoff value for that group was 0.278. Matches at the strain level also have been taken into account, when the centroid of tested specimen and the closest centroid in database were obtained for one and the same strain. The rate of these matches was 33.3% for JC and Biotyper. For the Euclidean distance it was 20.8%.

## Discussion

Protein profiling using MALDI TOF mass spectrometry proved to be reliable for identification of closely related species, including *Bacillus* spp.^28^,^29^,^30^,^31^. However, it is currently restricted by the absence of free databases and software, and by the fact that commercial databases are mostly oriented towards strains encountered in clinical practice. New data can be added to these databases only by the manufacturer. Therefore, creation of open platforms and databases is an urgent task.

This goal requires a simple and reliable method with high fidelity that allows one to use a broad spectrum of analysis techniques. In this study, we propose an algorithm for representing mass spectra as vectors in a multidimensional Euclidean space. Many geometric methods can be used with this algorithm. We chose Euclidean distance as the simplest and most straightforward approach for the Euclidean space. As an alternative, we also implemented Jaccard coefficients, which allow one to calculate spectra similarity. In addition, 1 − *JC* is a geometric distance, which makes it suitable for such geometric methods as PCo.

As a model for testing this approach, we used 24 closely related strains of the *B. pumilus* group from the collection of the Institute of Cytology and Genetics SB RAS, which had over 98% sequence similarity for the 16S rRNA gene. The studied strains were isolated from various extreme habitats in various regions of Russia, including thermal springs, saline lakes, complex ore deposits, etc. For these species we found characteristic peaks on mass spectra and characteristic nucleotide substitutions in the 16S rRNA gene. 16S rRNA sequences were used to validate the results of mass spectrometry analysis. The mass spectra centroids of the studied strains were separated into two groups, which was confirmed by the Welsh’s t-test, even if only the first coordinate of the PCo matrix (PCo1) are used. These groups correspond to the two clusters detected on the phylogenetic tree. The dendrogram constructed by the Ward method using all 23 coordinates also yields similar results. We performed an additional analysis of mass-spectrometry data using Biotyper 3.0 as an extra check. All three methods used allowed us to reliably distinguish between two groups that correspond to two species, *B. pumilus* (P) and *B. altitudinus* (A).

The obtained reference database was used for identification of the studied microorganisms by wet-lab experiments. Identification accuracy was 98% for JC and 100% for Euclidean distances. Biotyper 3.1 analysis had identification accuracy of 100% when using 2.0 as cutoff score, which is defined by Bruker as “secure genus identification, probable species identification”. The score of the unidentified KG16(3) strain for the JC analysis was located at the border of cutoff value for the A group. In this study we had separate cutoff values for each species, because the A group centroids were significantly more compact in the 1 − *JC* geometric space than the P group centroids (data not shown). So cutoff score for the A group was more stringent (0.278) than for the P group (0.158). If cutoff values were averaged for the two groups, JC algorithm had 100% identification accuracy. Also a higher rate of matches at strain level for JC than for the Euclidean distance may offer a better performance of this method for strain-level identification.

Therefore, we demonstrated that the approach proposed in this study is suitable for identification of closely related species based on their mass spectra using *B. pumilus* and *B. altitudinis* as a model. Theoretically representing mass spectra as vectors in the Euclidean space allows one to use virtually unlimited number of coordinates for each centroid, which enables us to use both peak lists and raw mass spectra describing the spectrum as a curve as source data. It will allow us to take peak form into consideration and abandon peak peaking algorithm. The centroids, Euclidean distances, and strain descriptions are available in the Internet: http://www.bionet.nsc.ru/mbl/database/database.html.

## Methods

### Strain description

From the collection of extremophilic microorganisms of ICiG SB RAS we selected 24 strains that were identified as belonging to the *B. pumilus* group based on morphological and biochemical characteristics. This group contained strains isolated from extreme environments form various regions of Russia, including thermal springs, saline lakes, complex ore deposits, etc (Table 1).

### 16S rRNA analysis

A fragment of the ribosomal 16S rRNA gene was amplified using universal bacterial primers 16S-8-f-B (5′-AGRGTTTGATCCT GGCTCA-3′) and 16S-1350-r-B (5′-GACGGGCGGTGTGTACAAG-3′) in a 30 microlitre volume on a MyCycler thermal cycler (BioRad) using TaqSE DNA polymerase (SibEnzyme, Novosibirsk) according to manufacturers’ instructions. Amplified products were purified using the PCR purification KIT (Fermentas). DNA sequencing was performed using the BigDye teminator 3.1 kit (Applied Biosystems) according to manufaturers’ instructions in the SB RAS Genomics Core Facility. Sequences were analyzed and edited using the BioEdit program (http://www.mbio.ncsu.edu/BioEdit/bioedit). The following type strain sequences were used for phylogenetic analysis: *B. pumilus* (AY456263), *B. altitudinis* (AJ831842), and *B. safensis* (AB681259); *B. licheniformis* (EF433410) was used as an outgroup. Sequences were aligned by ClustalW2^32^ and phylogenetic trees were constructed using the Maximum Likelihood algorithm^33^ implemented in MEGA 6.0^34^. All 16S rRNA sequences obtained by us were deposited in GenBank (Table 1).

### Mass spectrometry analysis

Twelve separate colonies were taken for each strain. Colonies were transferred to 1.5 ml Eppendorf tube by a microbial transfer loop and resuspended in 300 microliters of deionized water. For inactivation of bacterial cells, 900 microliters of ethanol was added; cells were resuspended and sedimented by centrifugation for 2 min at 15600 g. Supernatant was removed and the sediment was dried for 5 min in an Eppendorf vacuum concentrator. Bacterial cell walls were destroyed by the addition of 50 microliters of 70% formic acid. Proteins were extracted by the addition of 50 microliters of acetonitrile followed by vigorous vortexing. The mixture was centrifuged for 2 min at 15600 g, and the supernatant was transferred into a clean tube for mass spectrometry analysis.

For mass spectrometry analysis, 1 microliter of protein extract was transferred to a stainless steel plate and allowed to dry at room temperature. Afterwards, 1 microliter of matrix (6 mg/ml of *α*-cyano-4-hydroxy-cinnamic acid in acetonitrile/water/trifluoroacetic acid solution (50:47.5:2.5, v/v)) was added. Spectra were obtained using an Ultraflex III MALDI TOF/TOF mass spectrometer (Bruker Daltonics) in the linear positive mode with laser frequency of 100 Hz in the 2000–20000 Da mass range. The voltage at the accelerating electrode was 25 kV; IS2 voltage, 23.45 kV; lens voltage, 6 kV; no extraction delay was made.

For each colony we obtained three spectra by summing 500 laser pulses (5 × 100 pulses from various positions of the target cell). Calibration was performed using *Escherichia coli* proteins: RL36 - 4365.3 Da, RS22 - 5096.8 Da, RL34 - 5381.4 Da, RL32 - 6315.0 Da, RL29 - 7274.5 Da, RS19 - 10300.1 Da.

A total of 36 spectra were obtained for each strain. Visual inspection was performed for all spectra in addition to computer analysis.

### Phyloproteomic analysis of mass spectrometry data

Flattening, baseline extraction, and peak picking for the obtained mass spectra were performed using mMass^35^ (www.mmass.org).

Mass lists obtained in mMass were processed using the following algorithm:

A set of spectra is represented as {(*x*_*j*_, *y*_*j*_), *j* = 1…*L*_*m*_; *m* = 1…*M*}, where *x*_*j*_ is the peak number, *y*_*j*_ is the function of signal intensity, *L*_*m*_ is the number of peaks for each spectrum, *M* is the number of spectra. Spectra are divided into *Q* classes.

Each spectrum is projected on a grid uniform in the x-coordinate. For each spectrum, the grid has the same boundaries (*X*_*beg*_, *X*_*end*_), numbers of points *N*, and window half width K measured in points. The parameters *X*_*beg*_, *X*_*end*_, *N*, and *K* are set by the user. Grid spacing *h* and half-width size *w* are calculated based on input parameters:


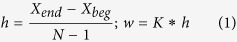


The following condition must be met for each spectrum: *X*_*beg*_ < *X*_*min*_ − *w*; *X*_*end*_ > *X*_*max*_ + *w*, where *X*_*min*_ and *X*_*max*_ are the minimum and maximum values for the x-coordinate for each spectrum.

For each mesh point *i* we find all x-coordinates *x*_*j*_ in the [*ih* − *w*, *ih* + *w*] range with nonzero *y*_*j*_ signal intensities. For each framed signal its impact on the point *i* is calculated by the formula:


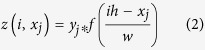


where *f*(*x*) = 1 − 3*x*^2^ + 2|*x*|^3^ when |*x*| ≤ 1 and *f*(*x*) = 0 otherwise.

The summarized impact *z*_*i*_ on point *i* is calculated by the formula:


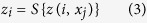


for each *x*_*j*_ within the window [*ih* − *w*, *ih* + *w*], where *S* is the combination method (sum, averaging, maximum). As the result of this algorithm, a vectors sized *N* was obtained for each spectrum.

In this work we used the following parameters: *X*_*beg*_ = 1000; *X*_*end*_ = 15000; *N* = 14000; *K* = 5; combination method was averaging.

When spectra were projected on the grid, centroids for every of *Q* classes were calculated. A matrix of Euclidean distances between all centroids was computed and processed by the Principal Coordinates analysis (PCo), which allowed us to represent all class centroids as points in a multidimensional Euclidean space with dimension no more than *Q* − 1. Welsh’s t-criterion was used to validate the studied groups on the PCo plot. Dendrogram were built using the Ward’s method by the PAST program^36^.

Jaccard coefficients are calculated according to the formula^37^:


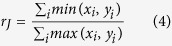


where *x* = {*x*_*i*_ : *x*_*i*_ ≥ 0}, *y* = {*y*_*i*_ : *y*_*i*_ ≥ 0} are vector representations of spectra.

Peak frequencies were analyzed using SPECLUST with the ≪peak in common≫ procedure. Search was performed in the 2 to 10 kDa range, which provides the optimum peak reproducibility with the “Width in peak match score” parameter value of 5 Da. In addition, the spectra were analyzed visually.

In addition, “main spectra” (MSPs) were generated in Biotyper 3.1 for the obtained mass spectra, and a phyloproteomic diagram was built using standard parameters^18^.

### Wet-lab experiment

For wet-lab experiments all studied strains were grown under the same conditions as for the training sample (Table 1). For mass spectrometry we took three replicates for each specimen, one spectrum per each replicate. Specimen centroids were calculated as described in the Phyloproteomic analysis of mass-spectrometry data section.

The aim of the experiments was to assign each tested specimen either to A or P groups, or to a separate species. Cutoff radius of the group *i* (A or P) were calculated using the following formula^38^:


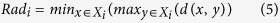


where *X*_*i*_ is the set of *i* centroids; *d*(*x*, *y*) is the distance between spectra *x* and *y*.

A centroid of tested specimen belongs to the “attraction zone” of group *i* if its distance from at least one centroid of this group does not exceed *Rad*_*i*_. If specimen belong to one or more “attraction zones” of different groups *i* its assign to the group of the closest centroid. If a specimen does not belong to “attraction zone” of any group in the database, it is treated as an unknown species.

## Additional Information

**How to cite this article**: Starostin, K. V. *et al.* Identification of *Bacillus* strains by MALDI TOF MS using geometric approach. *Sci. Rep.* **5**, 16989; doi: 10.1038/srep16989 (2015).

## Figures and Tables

**Table 1:** List of the studied strains.

Strain	Cultivation Medium	GenBank	Strain source	Geochemical characteristics	Coordinates
O48	LB	KP699776	NR, Solenoye l. (48). Water sample.	190; 15–20; 8, 0	54°14′*N*78°13′*E*
O32	LB	KP699772	NR, Gorkoye l. (42). Bottom sediments.	280; 15–20; 7, 7	54°17′*N*77°27′*E*
O19	LB	KP699775	NR, Dolgoye l. (44). Water sample.	43; 15–20; 8, 3	54°10′*N*77°56′*E*
O6	LB	KP699774	NR, Solenoye l. (48). Water sample.	190; 15–20; 8, 0	54°14′*N*78°13′*E*
47(6)il	S4	KP699765	NR, Khorosheye l. (47). Bottom sediments.	99; 15–20; 9, 2	54°05′*N*77°51′*E*
51(3)w	LB	KP699766	NR, Gorkoye l. (51). Water sample.	49; 15–20; 8, 9	54°12′*N*77°02′*E*
O41	LB	KP699767	NR, Krugloe l. (45). Bottom sediments.	290; 15–20; 7, 7	54°08′*N*77°56′*E*
O33	LB	KP699768	NR, Gorkoye l. (42). Bottom sediments.	280; 15–20; 7, 7	54°17′*N*77°27′*E*
48(1)w	LB	KP699778	NR, Solenoye l. (48). Water sample.	114; 15–20; 8, 0	54°14′*N*78°13′*E*
46(5)il	LB	KP699764	NR, Razboynoye l. (46). Bottom sediments.	14, 7; 15–20; 8, 7	54°07′*N*77°55′*E*
51(1)il	LB	KP699773	NR, Gorkoye l. (51). Bottom sediments.	133; 15–20; 8, 0	54°12′*N*77°02′*E*
51(5)il	LB	KP699777	NR, Gorkoye l. (51). Bottom sediments.	49; 15–20; 8, 9	54°12′*N*77°02′*E*
42(6)w	LB	KP699769	NR, Gorkoye l. (42). Water sample.	160; 15–20; 7, 6	54°17′*N*77°27′*E*
3U	MPA	KP699770	KR, A swamp near t. Ursk. Bottom sediments.	N.D.; 25; 2, 6	54°27′*N*85°24′*E*
10U	MPA	KP699771	KR, River Ur near t. Ursk. Water sample.	N.D.; 18; 7, 7	54°27′*N*85°24′*E*
KH6	MPA	KP699782	KT, Geyser valley, G-16 (thermal cauldron).	N.D., 62; 3, 6	54°25′*N*160°7′*E*
KU3-5(2)	MPA	KP699787	KT, Uzon caldera, U3-5 (Oil field).	0, 4; 79; 4, 7	54°30′*N*160°0′*E*
KH2	MPA	KP699786	KT, Uzon caldera, U3-4-8 (Oil field).	1, 1; 63; 2, 9	54°30′*N*160°0′*E*
KG16(2)	MPA	KP699780	KT, Geyser valley, G-16 (thermal cauldron).	N.D., 62; 3, 6	54°25′*N*160°7′*E*
K6dt	MPA	KP699783	KT, Uzon caldera, U-4 (Bannoye lake).	0, 2; 36; 4, 6	54°30′*N*160°0′*E*
KH3	MPA	KP699785	KT, Uzon caldera, Uskv2.	0, 3; 95; 8, 4	54°30′*N*160°0′*E*
KG16(3)	MPA	KP699781	KT, Geyser valley, G-16 (thermal cauldron).	N.D., 62; 3, 6	54°25′*N*160°7′*E*
Cd1	MPA	KP699779	RG, Novosibirsk water storage basin.	<1, 0; 21; N.D.	55°04′*N*82°92′*E*
Cu1	MPA	KP699784	RG, Novosibirsk water storage basin.	<1, 0; 21; N.D.	55°04′*N*82°92′*E*

NR, Novosibirsk region, saline lakes (l.); KR, Kemerovo region, complex ore deposits; KT, Kamchatka thermal springs; RG, rhizosphere of the water hyacinth, Novosibirsk water storage basin. For mass spectrometry, bacterial strains were grown at 37 °C on the following agar media: Luria-Bertani broth (LB); meat-peptone agar (MPA), and S4 medium containing 1 g/l NaCl, 5 g/l MgCl2, 1 g/l KCl, 1 g/l CaCl2, 4 g/l tripthone, 2 g/l yeast extract. Geochemical characteristics of the source environments: salinity (g/l); temperature (C); pH.
